# Reconsidering the Use of the Mindset Assessment Profile in Educational Contexts

**DOI:** 10.3390/jintelligence9030039

**Published:** 2021-08-04

**Authors:** Alexander P. Burgoyne, Brooke N. Macnamara

**Affiliations:** 1 School of Psychology, Georgia Institute of Technology, 654 Cherry St., Atlanta, GA 30332, USA; 2 Department of Psychological Sciences, Case Western Reserve University, 11220 Bellflower Road, Cleveland, OH 44106-7123, USA; bnm24@case.edu

**Keywords:** mindset, implicit theories, mindset assessment profile, validity, reliability

## Abstract

The Mindset Assessment Profile is a popular questionnaire purportedly designed to measure mindset—an individual’s belief in whether intelligence is malleable or stable. Despite its widespread use, the questionnaire appears to assess an individual’s need for cognition and goal orientation more than mindset. We assessed the reliability, construct validity, and factor structure of the Mindset Assessment Profile in a sample of 992 undergraduates. The reliability of the Mindset Assessment Profile was questionable (α = .63) and significantly lower than the reliability of the Implicit Theories of Intelligence Questionnaire (α = .94), an established measure of mindset. The Mindset Assessment Profile also lacked convergent and discriminant validity. Overall scores on the Mindset Assessment Profile correlated significantly more strongly with need for cognition than with mindset. Item-level analyses supported this finding: most items correlated weakly or not at all with mindset, and correlated significantly more strongly with need for cognition and learning goal orientation. Exploratory factor analysis indicated that three factors were underlying scores on the Mindset Assessment Profile: need for cognition, mindset, and performance goal orientation. Based on its questionable reliability and poor construct validity, we do not recommend that researchers and educators use the Mindset Assessment Profile to measure mindset.

## 1. Reconsidering the Use of the Mindset Assessment Profile in Educational Contexts

*Mindset* refers to people’s beliefs about the nature of their abilities. People with a growth mindset believe that attributes such as intelligence can be developed, whereas people with a fixed mindset believe that these attributes are stable. Some researchers have argued that a growth mindset is beneficial for academic achievement, on the premise that students with a growth mindset will pursue challenges and be resilient to setbacks (Blackwell et al. 2007). However, recent empirical evaluations have revealed only weak evidence for most of mindset’s premises, including its relationships with goal orientation, pursuit of challenges, resilience to setbacks, and academic achievement (Burgoyne et al. 2020; Li and Bates 2019; Payne et al. 2007; Sisk et al. 2018). Nevertheless, educators frequently use growth mindset interventions to encourage students to adopt a growth mindset in an effort to improve their academic performance (Boaler 2013; Sisk et al. 2018).

Mindset interventions are a booming industry in the educational sector. For example, the for-profit company Mindset Works has sold growth mindset interventions products to parents, teachers, and schools for over a decade (https://www.mindsetworks.com/programs/ (accessed on 6 April 2021)). Included in these intervention programs is an eight-item measure—the Mindset Assessment Profile—which is used as a diagnostic tool to determine whether students have a growth or fixed mindset; that is, whether they believe intelligence is malleable or stable (Hall 2016; Thomas 2018). The Mindset Assessment Profile questionnaire is also provided on the company’s website, where visitors are encouraged to “Take the Mindset Assessment to Learn More About Your Mindset.” (http://blog.mindsetworks.com/what-s-my-mindset (accessed on 6 April 2021)). As a result, researchers and educators frequently administer the Mindset Assessment Profile to measure mindset in educational contexts (see, e.g., Bedford 2017; Cartwright and Hallar 2018; Hall 2016; Lim et al. 2020; Neufville 2019; Saia 2017; Thomas 2018; Wakefield 2019; Wolferd 2020). Often, the Mindset Assessment Profile is administered before and after an intervention to test whether it altered students’ mindsets. A change from pre- to post-intervention is taken as evidence that the intervention was successful (Bedford 2017; Cartwright and Hallar 2018; Saia 2017; Thomas 2018; Wolferd 2020), a point we return to in the Discussion. 

Given the Mindset Assessment Profile’s extensive use among researchers, educators, and students, the psychometric qualities of this scale have practical significance. Although Mindset Works describes the Mindset Assessment Profile as a “diagnostic tool drawn from research-validated measures” (http://blog.mindsetworks.com/what-s-my-mindset (accessed on 6 April 2021)), they do not provide any information on the reliability, construct validity, or factor structure of the scale. 

## 2. Present Study

We noticed that many items in the Mindset Assessment Profile appeared to be tapping constructs other than mindset, namely goal orientation—one’s drive to master new material and demonstrate competency (Elliot and Church 1997), and need for cognition—one’s tendency to engage in and enjoy thinking (Cacioppo and Petty 1982). If this is the case, students, teachers, and parents may have a misconstrued understanding of their mindset based on their Mindset Assessment Profile scores. 

According to mindset theory, goal orientations are related to one’s mindset of intelligence, but they are distinct constructs (Dweck and Leggett 1988). That is, individuals with a growth mindset are hypothesized to endorse learning goals, reflecting a desire to acquire new skills, whereas people with a fixed mindset are hypothesized to endorse performance goals, reflecting a desire to prove their abilities (or not demonstrate a lack of ability). Despite these claims, however, evidence suggests that mindset is only weakly related to goal orientation. For example, in a sample of 438 undergraduate students, Burgoyne et al. (2020) found that mindset was weakly correlated with learning goal orientation (*r* = .10) and performance goal orientation (*r* = −.11), and Payne et al. (2007) found correlations of a similar magnitude in meta-analytic work. In light of their results, Payne et al. (2007) concluded that the relationship between mindset and goal orientation had been overstated by proponents of mindset theory: “Contrary to Dweck’s (1986) perspective, the effect sizes were very small, providing little evidence for Dweck’s (1986) view that implicit theories are the primary underlying antecedent of GO [goal orientation]” (p. 140).

Need for cognition, on the other hand, is a relatively unexplored construct within mindset’s nomological network. At a conceptual level, one might expect that individuals with more of a growth mindset would rate higher on need for cognition. That is, mindset theory would likely predict that individuals with a growth mindset would enjoy mental challenges, such as thinking about complex problems, on the basis that they might learn from them. The empirical evidence for this relationship is scarce, but suggests a weak correlation. For example, Birney et al. (2018) found that growth mindset correlated *r* = .12 with need for cognition in a sample of 142 experienced business managers. 

A related concern is that researchers using the Mindset Assessment Profile may make inaccurate assumptions about the relationship between mindset and measured outcomes (e.g., academic achievement) if the Mindset Assessment Profile includes items measuring other non-mindset constructs. For instance, if Mindset Assessment Profile scores are contaminated by the inclusion of goal orientation items, the observed relationship between Mindset Assessment Profile scores and goal orientation will be exaggerated. As another example, if goal orientation and need for cognition are stronger predictors of academic achievement than mindset is, then the relationship between Mindset Assessment Profile scores and academic achievement will be artificially inflated due to the inclusion of items tapping these constructs. 

### Analyses

The purpose of this study was to assess the internal consistency reliability, construct validity, and factor structure of the Mindset Assessment Profile as a measure of mindset. We estimated the internal reliability of the Mindset Assessment Profile by computing Cronbach’s alpha (α; Cronbach 1951; see George and Mallery 2003, for rules of thumb for interpreting Cronbach’s alpha) and McDonald’s omega coefficient (ω; McDonald 1999; McNeish 2018; Zinbarg et al. 2005). Cronbach’s alpha tests for consistency among items within a measure, and McDonald’s omega indicates the proportion of variance in the scale scores accounted for by a single factor (Zinbarg et al. 2005).

Construct validity is the degree to which a measure’s variance is attributable to variance in the construct it is intended to measure rather than some other factor (O’Leary-Kelly and Vokurka 1998). Construct validity is evaluated in terms of convergent and discriminant validity. Convergent validity is the degree to which different measures designed to assess the same construct correlate with one another (Cunningham et al. 2001): measures of the same construct should be strongly related. We tested for convergent validity by correlating scores on the Mindset Assessment Profile with a well-established measure of mindset, Dweck’s (2000) Implicit Theories of Intelligence Questionnaire, which has been shown to have sound psychometric properties (Dweck 2000). Discriminant validity, on the other hand, refers to the extent to which measures designed to assess different constructs correlate with one another (Campbell and Fiske 1959). Compared with two measures assessing the same construct, measures designed to assess different constructs should be more weakly correlated. We tested for discriminant validity by correlating scores on the Mindset Assessment Profile with measures of need for cognition and goal orientation.

Finally, we conducted an exploratory factor analysis on the items in the Mindset Assessment Profile to assess its factor structure. If all or most items load well onto a single factor, this suggests the Mindset Assessment Profile is measuring a single personality construct. If items load better on multiple factors, this suggests multiple personality constructs are underlying scores on this measure.

## 3. Method

Methods were pre-registered at https://osf.io/N82F4/. 

### 3.1. Participants

The participants were 998 undergraduate students at Michigan State University, ranging in age from 18 to 31 (*M* = 19.73, *SD* = 1.48). Approximately 63% of the participants were female. Around 38% of the participants were in their first year of college, 28% were in their second year, 21% were in their third year, and the remaining 13% were in their fourth or fifth year. Six participants were excluded because they did not reach the end of the survey, leaving a final sample of 992 participants. Missing data (<1% of cases) were handled using listwise deletion on an analysis-by-analysis basis. With 992 participants, we had 89% power to detect significant correlations of *r* ≥ .10 (Faul et al. 2007). All participants provided informed consent and received partial course credit for their participation in the study. 

### 3.2. Measures

**Demographics.** Participants were asked to report their age, year in college, and gender.

**Mindset Assessment Profile.** Participants responded to the eight items in the Mindset Assessment Profile taken from the Mindset Works website (http://blog.mindsetworks.com/what-s-my-mindset (accessed on 6 April 2021)) using a six-point Likert scale ranging from “Disagree a lot” to “Agree a lot.” The items are listed in order of administration: (1) “No matter how much intelligence you have, you can always change it a good deal”; (2) “You can learn new things, but you cannot really change your basic level of intelligence”; (3) “I like my work best when it makes me think hard”; (4) “I like my work best when I can do it really well without too much trouble”; (5) “I like work that I’ll learn from even if I make a lot of mistakes”; (6) “I like my work best when I can do it perfectly without any mistakes”; (7) “When something is hard, it just makes me want to work more on it, not less”; and (8) “To tell the truth, when I work hard, it makes me feel as though I’m not very smart.” Even numbered items were reverse scored. The final score was the mean response to the items. 

**Mindset.** Participants completed Dweck’s (2000) Implicit Theories of Intelligence Questionnaire as a measure of mindset. Participants responded to eight items using a seven-point Likert scale, rating the degree to which they agreed or disagreed with each statement: (1) “You have a certain amount of intelligence, and you can’t really do much to change it”; (2) “No matter who you are, you can significantly change your intelligence level”; (3) “Your intelligence is something about you that you can’t change very much”; (4) “You can always substantially change how intelligent you are”; (5) “To be honest, you can’t really change how intelligent you are”; (6) “No matter how much intelligence you have you can always change it quite a bit”; (7) “You can learn new things, but you can’t really change your basic intelligence”; and (8) “You can change even your basic intelligence level considerably.” Odd numbered items were reverse scored. The response options ranged from “Strongly agree” to “Strongly disagree.” The final score was the mean response to the items. Higher scores on this measure correspond to more of a growth mindset, reflecting the belief that intelligence is malleable. Lower scores correspond to more of a fixed mindset, reflecting the belief that intelligence is stable. 

**Need for Cognition.** Participants completed Cacioppo et al.’s (1984) Need for Cognition Questionnaire. Participants responded to eighteen items using a seven-point Likert scale, rating the degree to which they agreed or disagreed with statements such as “I would prefer complex to simple problems” and “Thinking is not my idea of fun” (reverse scored). The response options ranged from “Strongly agree” to “Strongly disagree.” The final score was the mean response to the items. Higher scores correspond to greater need for cognition.

**Goal Orientation.** Participants completed an adapted version of Elliot and Church’s (1997) Goal Orientation Questionnaire. Participants responded to sixteen items using a seven-point Likert scale ranging from “Disagree a lot” to “Agree a lot.” This questionnaire assesses three goal orientations: learning goal orientation, performance approach goal orientation, and performance avoidance goal orientation. Participants rated the degree to which they agreed or disagreed with learning goal statements such as “I want to learn as much as possible,” performance approach goal statements such as “I strive to demonstrate my ability relative to others,” and performance avoidance goal statements such as “I worry about the possibility of performing poorly.” The final score for each goal orientation was the mean response to the items. Higher scores correspond to greater endorsement of each goal orientation.

**Procedure.** Participants completed the questionnaires online on Qualtrics. Participants were first presented with the three-item demographic questionnaire. The order of the remaining questionnaires was randomized across participants to control for potential order effects.

## 4. Results

Analyses were preregistered at https://osf.io/N82F4/ and were conducted using SPSS. Data are openly available at https://osf.io/N82F4/.

### 4.1. Reliability of the Mindset Assessment Profile

**Figure 1:**
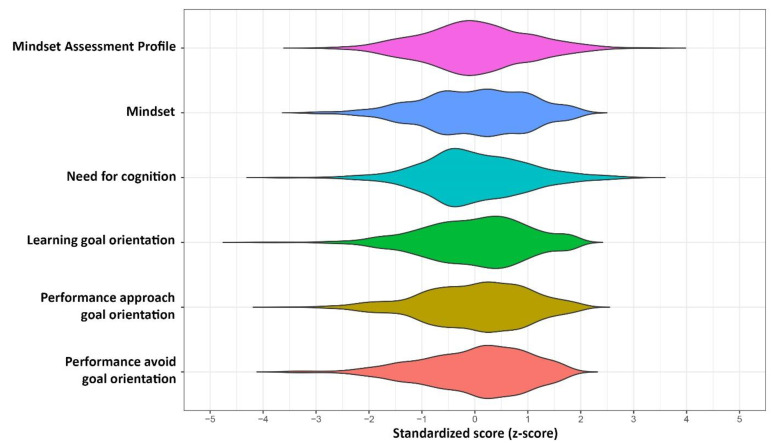
Distribution of standardized scores (i.e., *z*-scores) on each measure. *Note:* “Mindset” refers to the Implicit Theories of Intelligence Questionnaire.

Descriptive statistics are presented in Table 1 and distributions are presented in Figure 1. Of the questionnaires administered to participants, the Mindset Assessment Profile had the lowest reliability (α = .63, ω = .61). Table A1 in the Appendix A presents the inter-item correlations for the Mindset Assessment Profile. The correlations between the items varied in absolute magnitude from *r* = .05 to *r* = .49. Compared to the Mindset Assessment Profile, the Implicit Theories of Intelligence Questionnaire (“Mindset” in Table 1) had excellent reliability (α = .94, ω = .94). Indeed, the Mindset Assessment Profile had a significantly lower Cronbach’s alpha reliability estimate than the Implicit Theories of Intelligence Questionnaire, *t* = 38.89, *p* < .001 (Abd-El-Fattah and Hassan 2011). The other questionnaires had acceptable to good reliability, ranging from α = .72 to α = .87 (ω also ranged from .72 to .87).

### 4.2. Construct Validity of the Mindset Assessment Profile

**Figure 2:**
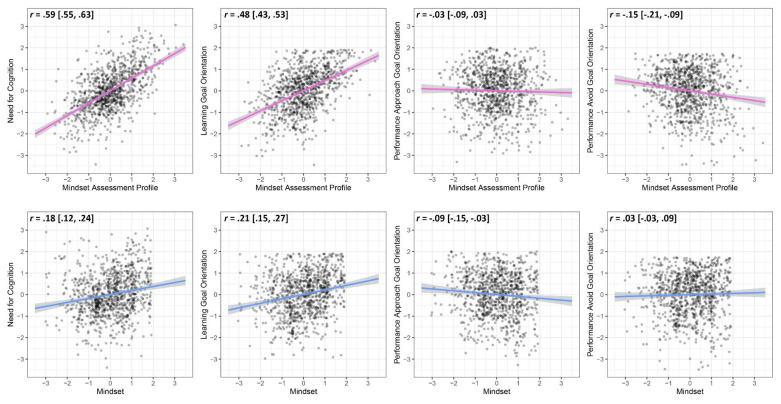
Scatterplots depicting correlations between Mindset Assessment Profile scores and other measures (top row) and between mindset and other measures (bottom row). *Note.* “Mindset” refers to the Implicit Theories of Intelligence Questionnaire. All scores are standardized (i.e., *z*-scores).

Correlations between measures are presented in Table 2. Scatterplots depicting the relationships between the Mindset Assessment Profile and the other measures (upper row) and between the Implicit Theories of Intelligence Questionnaire and the other measures (bottom row) are presented in Figure 2. Scores on the Mindset Assessment Profile correlated most strongly with need for cognition (*r* = .59, 95% CI [.55, .63], *p* < .001), followed by mindset (*r* = .50, 95% CI [.45, .55], *p* < .001) and learning goal orientation (*r* = .48, 95% CI [.43, .53], *p* < .001). Steiger’s (1980) test for the difference between dependent correlations revealed that the correlation between the Mindset Assessment Profile and need for cognition was significantly stronger than the correlation between the Mindset Assessment Profile and mindset, *z* = 2.87, *p* = .004. This indicates that the Mindset Assessment Profile lacks construct validity due to poor discriminant validity. Scores on the Mindset Assessment Profile were more closely related to need for cognition than mindset. For comparison, mindset as measured by the Implicit Theories of Intelligence Questionnaire correlated only weakly with need for cognition (*r* = .18, 95% CI [.12, .24], *p* < .001); see Figure 2. 

As an additional test of convergent and discriminant validity, we computed correlations between each of the items in the Mindset Assessment Profile and the other personality measures.^1^ The purpose of this analysis was to understand which items in the Mindset Assessment Profile correlated more strongly with non-mindset constructs than with mindset.

As shown in Table 3, most of the items in the Mindset Assessment Profile correlated more strongly with need for cognition and learning goal orientation than with mindset. Only items one and two correlated strongly with mindset (*r* = .70, 95% CI [.67, .73], *p* < .001 and *r* = .71, 95% CI [.68, .74], *p* < .001, respectively). This is not surprising, as the wording of these items is nearly identical to the wording of items six and seven in the Implicit Theories of Intelligence Questionnaire. 

By contrast, items three, four, five, six, seven, and eight from the Mindset Assessment Profile correlated significantly more strongly with need for cognition (average *r* = .38) than with mindset (average *r* = .12); Steiger’s test for the difference between dependent correlations revealed *z*s ranging from 3.32 to 11.96, all *p*s < .001. Items three, four, five, six, seven, and eight from the Mindset Assessment Profile also correlated significantly more strongly with learning goal orientation (average *r* = .29) than with mindset (average *r* = .12); Steiger’s *z*s ranged from 2.07 to 9.90, all *p*s < .02. Items four and six from the Mindset Assessment Profile did not correlate significantly with mindset (*r*s = −.01, 95% CIs [−.07, .05], *p*s > .65). 

These results indicate that most of the items in the Mindset Assessment Profile lack both convergent and discriminant validity. Most items correlated weakly or not at all with mindset, and correlated significantly more strongly with need for cognition and learning goal orientation than with mindset. A correlation matrix with the items from all the personality scales is presented on the Open Science Framework: https://osf.io/N82F4/.

### 4.3. Factor Structure of the Mindset Assessment Profile

Finally, we conducted an exploratory factor analysis on the items in the Mindset Assessment Profile to determine whether a single personality factor or multiple personality factors were underlying scores on this measure. We used principal axis factoring with promax rotation to allow extracted factors to correlate, and extracted factors with Eigenvalues ≥1.0.

As shown in Table 4, three factors emerged from the exploratory factor analysis of the Mindset Assessment Profile. Items three, five, and seven had high loadings on the first factor; items one and two had high loadings on the second factor; and items four and six had high loadings on the third factor. Item eight did not load highly on any factor. Although subject to interpretation, the first factor appears to represent need for cognition, the second factor appears to represent mindset, and the third factor appears to represent performance goal orientation. These results suggest that the Mindset Assessment Profile is not a unidimensional measure of mindset, but rather that three factors underlie scores on this measure. 

## 5. Discussion

We assessed the reliability, construct validity, and factor structure of the Mindset Assessment Profile in a sample of 992 undergraduate students. The internal reliability of the Mindset Assessment Profile (α = .63) was significantly lower than that of the Implicit Theories of Intelligence Questionnaire (α = .94), which had excellent reliability. Both of these measures consist of eight items and were ostensibly designed to measure mindset.

Further, the Mindset Assessment Profile lacked construct validity as a measure of mindset. Overall scores on the Mindset Assessment Profile correlated significantly more strongly with need for cognition than with mindset. Item-level analyses supported this finding, revealing that six of eight items in the Mindset Assessment Profile correlated more strongly with both need for cognition ($\bar{r}$ = .38) and learning goal orientation ($\bar{r}$ = .29) than with mindset ($\bar{r}$ = .12). Only two of eight items from the Mindset Assessment Profile correlated strongly with mindset (*r*s = .70 and .71) as measured by the Implicit Theories of Intelligence Questionnaire. These items are nearly identical to items from the Implicit Theories of Intelligence Questionnaire. Finally, two of the eight items in the Mindset Assessment Profile had no association with mindset as measured by the Implicit Theories of Intelligence Questionnaire (*r*s = −.01, *p*s > .65).

Exploratory factor analysis revealed that three factors were underlying scores on the Mindset Assessment Profile. These factors appeared to represent need for cognition, mindset, and performance goal orientation. This corroborates the previous results by showing that the Mindset Assessment Profile is not a unidimensional measure of mindset. 

The Mindset Assessment Profile is marketed as a measure of mindset. That is, students are encouraged to use the Mindset Assessment Profile to “assess their mindsets” on the Mindset Works website (http://blog.mindsetworks.com/what-s-my-mindset (accessed on 6 April 2021)). After completing the questionnaire, they are emailed a description of their “current mindset.” Regardless of their results, they are directed to a webpage that sells growth mindset interventions ranging in cost from $20 per student to $7500 per school (https://www.mindsetworks.com/programs/ (accessed on 6 April 2021)). 

Perhaps of greater concern, the Mindset Assessment Profile is included as a diagnostic tool in some of Mindset Works’ growth mindset intervention programs. If the Mindset Assessment Profile is administered before and after a mindset intervention, change scores might be taken as evidence that an intervention successfully altered a student’s mindset, when this effect would be more accurately described as a change in need for cognition. 

This tendency to misconstrue Mindset Assessment Profile scores is not uncommon. As a case in point, Bedford (2017) administered the Mindset Assessment Profile to secondary school students before and after a growth mindset intervention “to evaluate the success of the growth mindset interventions” (p. 433). When Bedford (2017) found significantly different Mindset Assessment Profile scores following the intervention, this was interpreted as evidence that the “interventions put in place were successful in changing mindset towards a growth mindset” (p. 436). As another example, Wolferd (2020) recently administered the Mindset Assessment Profile to elementary school children to evaluate the effects of Mindset Works’ Brainology program. Based on their scores on the Mindset Assessment Profile following the intervention, they reported that “female students in the experimental group displayed a significant, positive change in mindset” (p. 49) suggesting that “Brainology was an effective intervention for female students” (Wolferd 2020, p. 51). The research presented herein suggests that the Mindset Assessment Profile is more a measure of need for cognition than mindset, and that its description as a “mindset” assessment has led to misunderstandings about the efficacy of mindset interventions. 

Relatedly, Lim et al. (2020) recently used the Mindset Assessment Profile as a diagnostic tool to assess college students in a work-study program. Based on their scores on the Mindset Assessment Profile, students were categorized into those with a “growth mindset” and those with a “fixed or unsure mindset” (p. 111). They found that “growth mindset” students’ work-study supervisors rated them more highly on problem solving and decision-making than students not categorized as having a “growth mindset.” However, based on our research, a more accurate conclusion would be that students with higher need for cognition are more likely to be rated higher on problem solving and decision-making than students lower in need for cognition.

In sum, despite the Mindset Assessment Profile’s stated purpose as a mindset assessment and diagnostic tool, our results indicate that it is a poor measure of mindset. We recommend researchers avoid using the Mindset Assessment Profile as a measure of mindset or as a diagnostic tool in educational contexts.

## Figures and Tables

**Table 1:** Descriptive statistics.

Measure	Items	*N*	*M*	*SD*	Skew	Kurtosis	α	ω
Mindset Assessment Profile	8	992	3.70	0.62	0.11	−0.02	.63	.61
Mindset	8	992	4.72	1.26	−0.31	−0.28	.94	.94
Need for cognition	18	991	4.37	0.79	0.08	0.46	.87	.87
Learning goal orientation	5	991	5.48	0.84	−0.45	0.30	.78	.78
Performance approach goal orientation	6	991	4.96	1.07	−0.42	−0.07	.85	.86
Performance avoidance goal orientation	5	991	5.39	0.98	−0.62	0.15	.72	.72

*Note.* “Mindset” refers to the Implicit Theories of Intelligence Questionnaire.

**Table 2:** Correlation matrix.

Personality Measure	1	2	3	4	5
(1) Mindset Assessment Profile	---				
(2) Mindset	**.50**	---			
(3) Need for cognition	**.59**	**.18**	---		
(4) Learning goal orientation	**.48**	**.21**	**.58**	---	
(5) Performance approach goal orientation	−.03	**−.09**	.06	**.27**	---
(6) Performance avoid goal orientation	**−.15**	.03	**−.12**	**.20**	**.38**

*Note.* Listwise *n* = 990. Correlation coefficients in bold are statistically significant at *p* < .05.

**Table 3:** Correlations between items in the Mindset Assessment Profile and personality measures.

Mindset Assessment Profile Item	Mindset	Need for Cognition	Performance Approach	Performance Avoidance	Learning Goal
(1) No matter how much intelligence you have, you can always change it a good deal.	**.70**	.10	-.02	.04	.17
(2) You can learn new things, but you cannot really change your basic level of intelligence.	**.71**	.14	−.06	.00	.13
(3) I like my work best when it makes me think hard.	.14	**.57**	.13	.01	.50
(4) I like my work best when I can do it really well without too much trouble.	−.01	**.32**	−.04	−.13	.15
(5) I like work that I’ll learn from even if I make a lot of mistakes.	.22	**.38**	−.02	−.06	.34
(6) I like my work best when I can do it perfectly without any mistakes.	−.01	**.22**	−.14	−.21	.09
(7) When something is hard, it just makes me want to work more on it, not less.	.12	**.43**	.06	−.13	.39
(8) To tell the truth, when I work hard, it makes me feel as though I’m not very smart.	.20	**.33**	.02	−.12	.28

*Note.* Listwise *n* = 971. The correlation coefficient in bold is the strongest correlation for each item. |*r*s| ≥ .07 are statistically significant at *p* < .05.

**Table 4:** Exploratory factor analysis of the Mindset Assessment Profile.

Mindset Assessment Profile Item	Factor 1	Factor 2	Factor 3
(1) No matter how much intelligence you have, you can always change it a good deal.	.21	**.56**	−.22
(2) You can learn new things, but you cannot really change your basic level of intelligence.	−.12	**.87**	.12
(3) I like my work best when it makes me think hard.	**.69**	−.04	.05
(4) I like my work best when I can do it really well without too much trouble.	.03	.00	**.72**
(5) I like work that I’ll learn from even if I make a lot of mistakes.	**.62**	.02	−.06
(6) I like my work best when I can do it perfectly without any mistakes.	.06	.00	**.62**
7) When something is hard, it just makes me want to work more on it, not less.	**.56**	−.01	.09
(8) To tell the truth, when I work hard, it makes me feel as though I’m not very smart.	**.25**	.16	.09
**Eigenvalue**	2.28	1.53	1.15

*Note.* Listwise *n* = 973. The coefficient in bold is the strongest factor loading for each item. Correlations between factors: Factor 1 with Factor 2 (*r* = .26); Factor 1 with Factor 3 (*r* = .25), Factor 2 with Factor 3 (*r* = .00).

## Data Availability

Data and preregistration are provided at the following Open Science Framework link: https://osf.io/n82f4/?view_only=f236b81a4d434711aff5db4b26f319a8.

## Note

1 This analysis was not pre-registered.

## Appendix A

**Table A1:** Correlation matrix with all items from the Mindset Assessment Profile.

Mindset Assessment Profile Item	1	2	3	4	5	6	7
(1) No matter how much intelligence you have, you can always change it a good deal.	---						
(2) You can learn new things, but you cannot really change your basic level of intelligence.	**.49**	---					
(3) I like my work best when it makes me think hard.	**.16**	**.07**	---				
(4) I like my work best when I can do it really well without too much trouble.	**−.09**	**.07**	**.20**	---			
(5) I like work that I’ll learn from even if I make a lot of mistakes.	**.23**	**.08**	**.41**	.05	---		
(6) I like my work best when I can do it perfectly without any mistakes.	**−.10**	**.07**	**.15**	**.47**	**.15**	**---**	
(7) When something is hard, it just makes me want to work more on it, not less.	**.16**	**.08**	**.41**	**.20**	**.34**	**.16**	---
(8) To tell the truth, when I work hard, it makes me feel as though I’m not very smart.	**.12**	**.20**	**.25**	**.11**	**.20**	**.12**	**.16**

*Note.* Listwise *n* = 973. Correlations in bold are statistically significant at *p* < .05.
